# Fixing Work, Not Workers: Leveraging Artificial Intelligence to Combat Burnout in Canadian Healthcare

**DOI:** 10.1177/08404704261420330

**Published:** 2026-02-09

**Authors:** Taylor Martin, Robert Maunder, Gillian Strudwick

**Affiliations:** 1 Institute of Health Policy, Management and Evaluation, Dalla Lana School of Public Health, University of Toronto, Toronto, Ontario, Canada; 2 Mechanical and Industrial Engineering, University of Toronto, Toronto, Ontario, Canada; 3 Institute of Medical Science, University of Toronto, Toronto, Ontario, Canada; 4 Department of Psychiatry, Mount Sinai Hospital, Toronto, Ontario, Canada; 5 7978 Centre for Addiction and Mental Health, Toronto, Ontario, Canada

## Abstract

Burnout among healthcare workers in Canada remains a critical challenge with implications for workforce retention, patient safety, and system sustainability. Traditional responses have often emphasized individual coping strategies rather than structural change. This article argues that Artificial Intelligence (AI) might offer new opportunities to address some of the organizational drivers of burnout. We outline three domains where AI may provide value: (1) enhancing the measurement and understanding of burnout, (2) strengthening workforce planning and operational decision-making, and (3) mitigating workplace risks through process redesign and automation. By shifting attention from “fixing workers” to “fixing work,” AI might be part of the “solution” to support healthier, more sustainable healthcare environments.

## Introduction

Burnout among healthcare workers remains a critical issue in Canada, with downstream effects on recruitment, retention, patient safety, and system sustainability. Despite the extensive literature available, many organizational responses have focused on wellness programs or individual coping strategies, while the structural drivers of burnout like workload, scheduling, documentation demands, and job insecurity remain insufficiently addressed.^1^ In the post-pandemic era, these stressors have intensified. Healthcare workers are contending with prolonged periods of systemic strain, persistent understaffing, increasing patient complexity, and moral distress tied to resource constraints.^2,3^ With a growing share of the workforce citing burnout or stress as the primary reason for leaving or considering changing jobs, new approaches are needed to identify, prevent, and mitigate burnout at the system level.^3^

Artificial Intelligence’s (AI) application to healthcare is broad and rapidly evolving, spanning remote monitoring, diagnostic and treatment advances, automation of administrative tasks, and operational planning.^4^ Machine learning algorithms enable pattern recognition in large datasets and have been applied to medical imaging to improve diagnostic accuracy and separately to medical data to identify patients at risk of deteriorating.^4,5^ Natural Language Processing (NLP) refers to the ability for human language to be understood and interpreted by computers. NLP has been used to create virtual assistants and ambient scribes.^4^ Predictive analytics (forecasting) is another domain of AI and has been used to anticipate patient volumes and inform staffing levels. While the potential benefits of AI are numerous this commentary focuses on applications of AI to the drivers of burnout.

## Understanding Burnout: The Causes and Consequences

**Figure 1.**
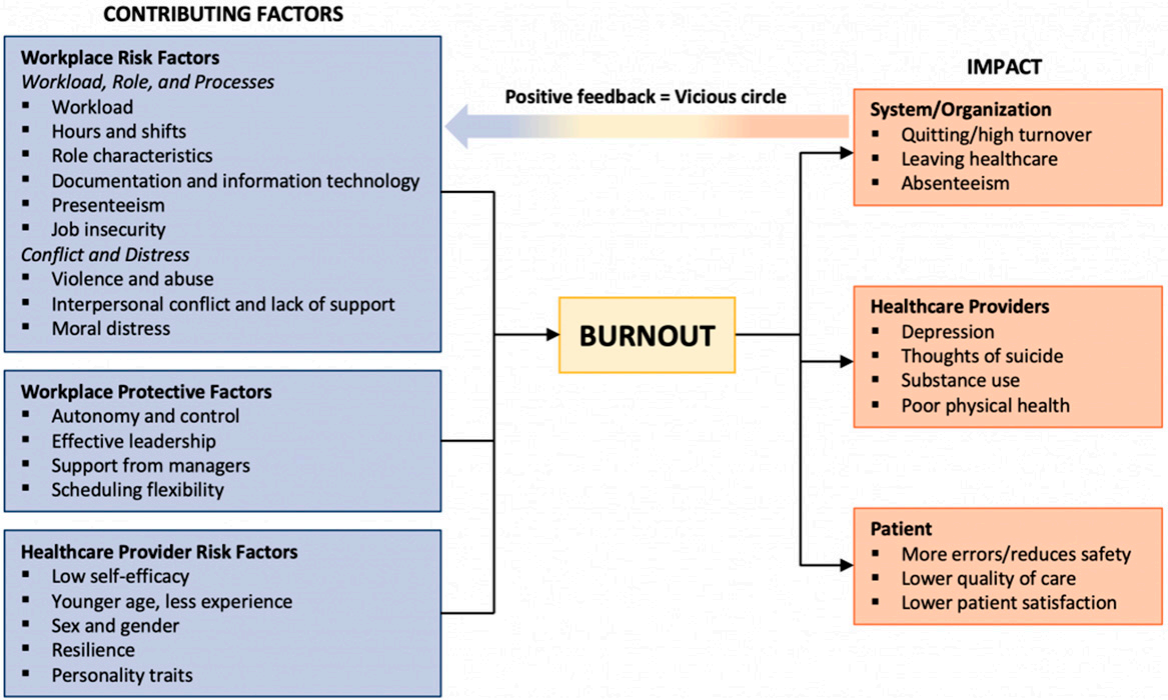
Causes and impact of burnout framework [from science table briefing note]

One framework that has been developed to understand the contributors to, and impact of, burnout was developed by members of the Ontario COVID-19 Science Advisory Table in 2021.^1^ The framework illustrates how organizational culture, workplace structures, and individual characteristics intersect to influence the risk of burnout among healthcare providers. It highlights that risk is rarely attributable to a single factor but instead emerges from the interplay of workload, role expectations, leadership, and personal resources such as self-efficacy and resilience. The framework organizes contributing factors into three broad domains: (1) workforce risk factors (e.g., workload, job insecurity, scheduling demands, and interpersonal conflict), (2) workforce protective factors (e.g., authentic leadership, autonomy, and scheduling flexibility), and (3) individual characteristics (e.g., age, gender, resilience, and personality traits) (see Figure 1).

While the framework highlights the complexity of burnout’s causes, it also points to an opportunity; many of these risk factors are observable, measurable, and potentially modifiable. This is where AI may offer new solutions which may become part of the solution to combatting burnout. Therefore, the purpose of this commentary is to explore how AI methods and tools might help address key drivers of burnout in Canadian healthcare. We argue that AI has the potential to: (1) enhance how burnout is measured and understood, (2) inform workforce planning and operational decisions, and (3) support prevention and mitigation of organizational causes of burnout, as identified in the framework described above.

## How AI Might Enhance How Burnout Is Measured and Understood

Measurement of factors contributing to burnout at the organizational and individual levels is critical to understanding and taking action. One opportunity is to create an “at risk for burnout” dashboard. This could mimic CHARTWatch, a partnership between Unity Health Toronto and Signal 1. CHARTWatch is a machine learning-based early warning system for patient deterioration trained on Electronic Medical Record (EMR) data.^5^ The tool is used to identify patients most at risk based on their latest metrics. Evaluations of CHARTWatch showed a 26% reduction in unexpected deaths with its use. An important clinical tool in its own right, CHARTWatch’s approach could be used to better understand burnout and focus intervention.

Just as CHARTWatch considers a range of clinical inputs in its forecast, machine learning models could be trained on “working-conditions” data to identify clinical areas, and indeed individuals, at highest risk of burnout. EMRs are rich with data related to nursing and other health discipline workload, such as acuity and patient volume. Scheduling data are a source for shift length and shift patterns that may contribute to fatigue and can be paired with EMR data to compute actual nurse to patient ratios. Scheduling data also tell us about scheduling flexibility (e.g., time off requests approved or denied), vacation taken, overtime, and staff “outcomes” such as unplanned absenteeism. Other data, such as staff engagement surveys or incident reports, speak to staff-reported elements of culture, working conditions, leadership support, and moral distress. Human Resources data on employment status (full time or part time, permanent or temporary) and, ultimately, turnover are key information for understanding burnout at the individual, unit, or organizational scale.

Such a data-driven “at risk for burnout” AI model could be used to guide interventions to reduce burnout. There are a number of workload measures widely used in practice to set staffing ratios. However, research shows different tools lead to very different recommendations.^6^ A learning health system could establish its own AI tool trained on its particular data to set and adjust staffing levels. Where indicators spike or trend out of control, leaders must identify short- and long-term changes that may benefit their teams. This challenge is aided by the availability of large, integrated data sets. When overtime is high, it may be due to recent sick time or could be a result of higher-than-normal acuity: two different causes calling for different responses. Excessive turnover could trigger the AI to suggest both additional staffing resources and focused support for seasoned nurses who will take on more while novice staff are onboarded. AI models built on linked data can quickly point to underlying causes.

AI tools for forecasting and classification are only as good as the data they are trained on. Time series data on acuity, nurse to patient ratio, and absenteeism are only available with modern, integrated information systems. Organizations that have invested in electronic health records, human resource information systems, and workforce management systems are well positioned to lead in this space. This does not mean that leaders must wait on modern systems entirely; building descriptive analytics tools using existing datasets (e.g., basic turnover and overtime data available in older systems) is an easy first step to establishing data literacy in leadership teams and building demand for more advanced solutions.

## How AI Might Inform Workforce Planning and Operational Decisions

AI and machine learning promise to accelerate effective workforce planning and operational decision-making by creating better forecasts. Data have always been the basis for workforce planning in healthcare; forecasts draw on traditional datasets such as headcount, turnover, and previous years’ patient bed days. AI forecasting can use big data approaches to incorporate vast (and varied) datasets in new ways. This could include disparate sets such as unstructured EMR data (deidentified) and regional census data from Statistics Canada to identify patterns and predict demand for certain types of services or care providers. Forecasts can address long-term (“how many should be trained?”), medium-term (“how many should be hired?”), and short-term (“how many should be scheduled?”) questions.

AI will also aid in the availability and pace of forecasting. Instead of creating a one-time forecast to plan from, AI and machine learning can rapidly re-forecast, improving their accuracy as they learn from more data. As weather forecasts for a long weekend change in the days leading up, unit managers can re-run a prediction of Emergency Department visits multiple times to update staffing requirements proactively. Anticipating changes in demand can mitigate short staffing, reduce overtime, and keep shifts to 12 hours or less, each a contributor to burnout.^1^

Even while an individual AI forecasts may improve decision-making, the availability of multiple AI forecasts can also enhance predictions. Previously, a single forecast may have informed workforce planning; now, multiple competing AI models can be produced for the same task. Ensemble Learning is an emerging approach to balance the inherent errors in any single model. Just as the collective accuracy of several human forecasts can outperform a single forecast, research has shown that multiple Large Language Models (LLMs) deployed in parallel to predict events can produce more accurate and robust average predictions than any one model alone.^7^ For a manager, a range of forecasts could help them select the best “average” decision while making contingency plans for the less likely but high-impact outcomes the models predicted.

Health leaders may also adopt AI for scenario planning. A leader considering which retention strategies or training opportunities to invest in could use AI tools to evaluate their relative impact. LLMs can assist experts in developing scenarios by asking questions to refine thinking, drawing on data to understand hard-to-quantify relationships, and providing preliminary evaluation by identifying strengths, risks, and implementation considerations.^8^ AI can be used to produce and adapt mathematical simulation models based on scenario characteristics, providing leaders with a more quantifiable understanding of the impacts of their decisions.

## How AI Might Support Prevention and Mitigation of Organizational Causes of Burnout

AI has the potential to reduce some of the organizational drivers of burnout by addressing inefficiencies and administrative burdens that weigh on healthcare providers. For example, AI-enabled “ambient documentation/scribes” and automated order set completion can streamline documentation and lessen the burden of using an electronic health record system.^9^ Similarly, automation of traditionally back-office processes, payroll, or supply chain management can reduce administrative load and improve workflow efficiency. In addition to automation, AI can learn from routine processes to identify and diagnose bottlenecks, as demonstrated in simulation models.^10^

SiMLQ is one healthcare simulation platform that leverages AI-driven digital twins and process mining to optimize patient flow and workforce allocation.^11^ Hospital event logs, such as patient arrivals, job types, and resource schedules, are used to generate predictive models of demand and simulate different staffing and policy scenarios in real time. AI-driven digital twins can enable health systems to anticipate workload peaks, test new scheduling approaches, and identify bottlenecks before they escalate into burnout-inducing pressure points for staff. Early applications in emergency and infusion clinic settings suggest that by reducing unpredictability and aligning staffing with demand, such tools can help lower administrative burden and support a more sustainable workload for frontline healthcare workers.^12^

Workforce scheduling is another area of potential high impact.^13^ AI-powered systems may assist in optimizing assignments to support safe workloads, reduce overtime, and incorporate staff preferences for shifts, which can enhance flexibility and autonomy.^1^ Mathematical optimization techniques can easily construct “optimal” schedules that meet operational and union requirements. Yet seasoned managers know that optimal becomes obsolete the moment an unexpected absence occurs. AI is particularly suited to assist in “rescheduling” activities, whether to re-optimize the schedule over many days as resources change or to rapidly re-do patient assignments at the start of shift.

AI systems may be particularly valuable for new clinical staff, who often experience a disconnect between training expectations and the realities of practice.^14^ The workload to care for a complex patient, as experienced by the nurse, may be higher for novice nurses.^14^ AI could be used to assist in patient assignment according to nurse tenure, skills, and acuity factors. By making work more predictable, balanced, and responsive to staff needs, AI tools may be able to help organizations address structural causes of burnout while building more sustainable and supportive work environments.

## Challenges and Considerations

The development of AI tools leveraging Personal Health Information (PHI) must consider ethical and privacy concerns. In the Canadian context, compliance with provincial legislation such as the Personal Health Information Protection Act (PHIPA) is essential, alongside clear governance for how PHI are accessed, used, and protected when training and deploying models. Additionally, AI systems may introduce the risk of error or unintended bias; therefore, appropriate guardrails which may include transparency, human oversight (e.g., “human in the loop”), and limits on secondary data use can be implemented to mitigate harm and maintain trust.

Technical and logistical barriers may hinder the application of AI to healthcare worker burnout. Home-grown solutions require a sophisticated development pipeline, from ensuring privacy while leveraging sensitive data to validating the accuracy of AI models.^5^ For persevering leaders, researchers like Verma et al provide practical guidance to explore potential applications, design solutions, and evaluate the impact.^15^ Technological limitations such as poor interoperability may also delay deployment of AI; the patchwork of enterprise software systems can include modern Software as a Service and legacy installations from different eras which may not be AI-ready by design.

## Implications for Health Leaders

For health leaders, the promise of AI lies less in revolutionary breakthroughs and more in the practical optimization of organizational processes that drive burnout, such as scheduling, workload management, documentation, and staffing planning. AI should not be seen as a “silver bullet” without the appropriate governance, transparency, and co-design with staff (and others affected by these tools); these tools risk worsening distrust and even adding to the pressures they aim to alleviate if inappropriately implemented. Health leaders should therefore prioritize thoughtful implementation, ensuring that AI systems are “workforce-aware,” contextually relevant, and aligned with organizational readiness in terms of technology, data infrastructure, and culture. Most importantly, embracing AI as a tool to “fix the work, not the workers” reframes burnout prevention as a shared organizational responsibility rather than an individual burden.

## Conclusion

Burnout remains a systemic challenge in Canadian healthcare which is demanding of solutions that extend beyond individual resilience. AI tools, when applied responsibly and appropriately, might actually help identify, predict, and mitigate the structural drivers of burnout while supporting workforce sustainability. By reorienting efforts toward organizational change, healthcare systems can move closer to creating environments where both healthcare providers and patients can thrive.
